# Relationship Between Economic Loss and Anxiety During the Coronavirus Disease 2019 Pandemic: Moderating Effects of Knowledge, Gratitude, and Perceived Stress

**DOI:** 10.3389/fpsyt.2022.904449

**Published:** 2022-06-13

**Authors:** Hyerim Jang, A-La Park, Yu-Ri Lee, Seunghyong Ryu, Ju-Yeon Lee, Jae-Min Kim, Sung-Wan Kim, Young-Shin Kang

**Affiliations:** ^1^Department of Psychology, Chonnam National University, Gwangju, South Korea; ^2^Care Policy and Evaluation Centre, Department of Health Policy, London School of Economics and Political Science, London, United Kingdom; ^3^Department of Social Welfare, Nambu University, Gwangju, South Korea; ^4^Department of Psychiatry, Chonnam National University Medical School, Gwangju, South Korea; ^5^Mindlink, Gwangju Bukgu Mental Health and Welfare Center, Gwangju, South Korea

**Keywords:** COVID-19, anxiety, economic loss, knowledge related COVID-19, gratitude, perceived stress

## Abstract

**Objectives:**

The prolonged coronavirus disease 2019 (COVID-19) pandemic has caused individuals to suffer economic losses, in particular due to the implementation of intensive quarantine policies. Economic loss can cause anxiety and has a negative psychological impact on individuals, worsening their mental health and satisfaction with life. We examined the protective and risk factors that can influence the relationship between economic loss and anxiety during the COVID-19 pandemic.

**Methods:**

Panel data from 911 participants were collected in April and May 2020 and again 6 months later. We analyzed the relationship between economic loss and anxiety and investigated the moderating effects of knowledge about COVID-19, gratitude, and perceived stress. Moreover, we investigated whether there were any changes in moderating effects over time or in different demographic groups.

**Results:**

In the early stages of the spread of COVID-19, gratitude (*B* = –0.0211, *F* = 4.8130, *p* < 0.05) and perceived stress (*B* = 0.0278, *F* = 9.3139, *p* < 0.01) had moderating effects on the relationship between economic loss and anxiety. However, after 6 months, only perceived stress had a significant moderating effect (*B* = 0.0265, *F* = 7.8734, *p* < 0.01).

**Conclusion:**

In the early stages of COVID-19, lower levels of gratitude and higher perceived stress led to greater anxiety. In later stages of the prolonged pandemic, only perceived stress had a continued moderating effect on the relationship between economic loss and anxiety. This study suggests that psychological interventions to reduce perceived stress are needed to treat the possible adverse effects of the spread of infectious diseases on mental health.

## Introduction

Coronavirus disease 2019 (COVID-19) is a novel infectious disease that has been prevalent worldwide since it was first confirmed in December 2019. Quarantine and isolation are essential to preventing the transmission of COVID-19. Therefore, policies to restrict the public lives of individuals, such as limiting public crowds, have been implemented globally.

Although social distancing is effective for preventing the spread of viruses (1), it brings about great economic loss. According to the Organization for Economic Co-operation and Development (OECD) (2), the real gross domestic product of the world decreased by 4.2% in 2020. Compared to 2019, the number of employed and self-employed individuals in South Korea decreased by 218,000 and 75,000, respectively, in 2020 (3). This indicates an increase in the number of individuals facing economic difficulties following the COVID-19 outbreak. Holmes et al. (4) suggested that the economic difficulties caused by COVID-19 quarantine policies can have serious mental health implications. Studies conducted in South Korea have demonstrated that individuals with reduced income due to COVID-19 have significantly higher anxiety compared to those with no reduction in income (5, 6). The OECD (7) reported that individuals who were unemployed or in unstable occupations in the United Kingdom during the COVID-19 crisis complained of higher levels of mental anguish. According to a review of psychological effects of large-scale epidemics (8), long quarantine periods prevent adequate provision of necessary information and supplies, which causes individuals to experience financial loss. This can add to the stress of fear of infection and may have negative psychological effects, such as anxiety, anger, confusion, and posttraumatic stress.

Anxiety is the most common symptom in unstable situations. Global uncertainty due to COVID-19 spread (9) was a persistent threat that could not be avoided (10). This reduced the overall quality of life during the spread of COVID-19 (11). According to the theory of uncertainty, which is related to generalized anxiety disorder, some individuals poorly tolerate the possibility of occurrence of a negative event, regardless of its probability (12). Therefore, it may be assumed that economic loss due to prolonged COVID-19 would make individuals extremely vulnerable to anxiety. In fact, low tolerance of the uncertainty of COVID-19 partially mediates adjustment disorders and causes generalized anxiety disorder (9). Moreover, studies in Korean (13, 14) and Chinese (15, 16) populations have reported substantial anxiety reactions. According to a Korean big data study (17), anxiety is one of the top keywords related to negative psychological effects of COVID-19, along with prolonged, lethargic, stress, and fear. Therefore, determining the factors that can influence the effects of economic loss due to COVID-19 on anxiety would be helpful for future psychotherapy and counseling interventions.

Fear and anxiety due to uncertainty were characteristic of the early stages of the pandemic, and unverified information propagated easily because there was limited information about the disease (18). In such a situation, false information about transmission, treatment, and prevention can exacerbate psychological problems (19). Moreover, like a lack of information, excessive information also increases anxiety (16, 20). Evidence suggests that excessive use of social media and consumption of information about COVID-19 can increase anxiety (16, 21, 22). Media may not deliver correct knowledge about COVID-19; rather, it may spread anxiety and fear due to unverified information (23). In contrast, providing adequate and accurate information about infectious diseases may reduce confusion and anxiety (23, 24). A nationwide mental health survey conducted by the Korean Society of Traumatic Stress Studies reported “information related to infectious diseases” as the most important information for the public (25). Therefore, accurate knowledge may regulate negative emotions caused by COVID-19, thereby acting as a protective factor.

Other protective factors that can reduce the effects of the pandemic, such as gratitude, can also be considered. Gratitude is a well-known concept in the field of positive psychology, and people with high levels of gratitude can find positive aspects even in negative situations and reinterpret events (26). Individuals with a lot of gratitude are highly satisfied with their lives, frequently experience optimistic or positive emotions, and have lower levels of depression and stress (27, 28). In studies related to COVID-19, gratitude were positively associated with mental wellbeing during the lockdown in the United Kingdom (29). Individuals with stronger religious beliefs have a greater tendency to be grateful, and religion positively affects psychological wellbeing (30, 31). Religious people are highly likely to use their religious beliefs to deal with uncertainty and alleviate anxiety during the COVID-19 pandemic. In severely stressful situations, such as difficulties with outdoor activities or economic loss due to the spread of an infectious disease, gratitude and religion seem to relieve stress-related anxiety.

Similarly, individual perceptions and subjective judgments of stress levels due to an event can also affect anxiety. In times of stress, individuals perceive their stress level based on their subjective evaluation of an event, their resources, and their capacity to control it instead of objective parameters (32). Anxiety and perceived stress have a bidirectional relationship. People who have anxiety disorders are more affected by stressful events (33). Conversely, stressful events tend to precede anxiety disorders (34). Therefore, individuals who perceive their stress levels to be high during an event may experience more emotional difficulties. A longitudinal study showed that Dutch adults who reported higher levels of perceived stress during the COVID-19 lockdown experienced greater negative emotional changes, such as anxiety and hostility (35).

As COVID-19 is becoming a “social disaster” because of its prolonged global effects, there is a great need for studies examining temporal changes (36, 37). Various studies have reported the actual psychological impact of COVID-19 ranging from anxiety, panic, and fear to more long-term distress such as PTSD, depression, and grief (38, 39) and have warned of the risk for neurological sequelae from headaches, olfactory and gustatory dysfunction, and sleep disturbance to cognition and memory complications (40). It may be important to determine protective and risk factors in terms of time and social demographics for such a long-term disaster. Therefore, we examined the moderating effects of knowledge about COVID-19, gratitude, and perceived stress on anxiety due to economic loss in the early stages of the pandemic in South Korea and whether these effects changed after 6 months. Moreover, by dichotomizing individual characteristics and analyzing each moderating effect model, we aimed to determine whether the model used in this study could have moderating effects within different groups.

## Materials and Methods

### Participants and Procedures

This study enrolled individuals ages 19–65 years who lived in metropolitan areas, such as Seoul and its surrounding areas, Daegu, and Gwangju, which are representative metropolitan cities. Quota sampling was used to ensure a uniform age and sex distribution within the regional groups. Two online questionnaires were used to assess change over time. The first questionnaire survey was conducted between April 24 and May 5, 2020, 3 months after the COVID-19 outbreak in South Korea; the second questionnaire survey was conducted between November 9 and 23, 2020, 6 months after the first survey. Data collection methods previously described for the general population were used (41). All participants were selected from the panels of an online survey service (Macromill Embrain, South Korea). The first data collection period (April–May 2020) included 1,500 participants. The second survey questionnaire was sent to the 1,500 participants who were sent the first questionnaire, and 60.7% of them responded. A total of 911 participants who answered both questionnaires were included in the final analyses. The study was approved by the Chonnam National University Hospital Institutional Review Board (CNUH-2020-092).

### Measurement

#### Sociodemographic Information

In this study, sex, age, religion, job type, and medical insurance type were considered variables for individual characteristics. The questionnaire presented various religions (e.g., Christianity, Buddhism), including “no religion,” but responses were recoded as only the presence or absence of religion. Job type (regular worker/long-term contract worker, short-term contract worker, or without any regular income) and medical insurance type were analyzed to assess socioeconomic status. As COVID-19 is a medical issue, it was relevant to assess whether an individual had medical insurance. The health insurance system in South Korea provides health insurance to all individuals who have been employed for more than a month, along with their dependents, and to all individuals who run a business (42). Medical aid is provided to the remaining low-income people who have difficulty maintaining a livelihood (42). Therefore, individuals provided with medical aid can be regarded the poorest people in South Korea.

#### Economic Loss

To measure COVID-19-related economic losses, we used previously described self-report questionnaires (41, 43). Two items for economic problems were selected to measure distress related to the COVID-19 outbreak: “In the aftermath of COVID-19, I have experienced a loss in income.” and “I am experiencing economic stress (increased economic burden due to less income or more inflation)” All items were assessed on a 5-point Likert scale ranging from “not at all” (1 point) to “very much so” (5 points). Higher scores represented greater difficulties with external activities and economic loss. Cronbach’s alphas were 0.811 and 0.793 for the first and second surveys, respectively.

#### Anxiety

The Generalized Anxiety Disorder scale (GAD-7; 44) was used to measure anxiety among study participants. This questionnaire, developed by Spitzer et al. (44), consists of seven items. Cronbach’s alphas were 0.925 and 0.922 for the first and second surveys, respectively.

#### Knowledge About Coronavirus Disease 2019

Knowledge about COVID-19 was assessed with a 6-item researcher-developed questionnaire (23) (Table 1). Higher scores indicated greater knowledge about COVID-19. The Kuder-Richardson Formula 20 for this scale, which corresponds to dichotomous questions (45), was 0.78 and 0.43 for the first and second surveys, respectively.

**TABLE 1:** Questionnaire to measure knowledge of COVID-19.

	Statements
Q1.	COVID-19 is spread through the saliva of infected people. (True)
Q2.	To prevent infection with COVID-19, it is necessary to avoid touching your eyes, nose or mouth with your hands. (True)
Q3.	Washing your hands under running water with soap for at least 30 s helps to prevent COVID-19 infection. (True)
Q4.	When coughing or sneezing, it is necessary to cover your mouth with your palm. (False)
Q5.	Windows should be kept closed as much as possible, as the virus can enter while ventilating a room. (False)
Q6.	COVID-19 is a fatal disease causing death in more than 30% of affected general adults. (False)

#### Gratitude

The Gratitude Questionnaire-6 (GQ-6), developed by McCullough et al. (27) and translated and validated for Koreans by Kwon et al. (46), was used. The GQ-6 is a self-report measure that evaluates the experience and expression of gratitude in daily life. Cronbach’s alphas were 0.894 and 0.892 for the first and second surveys, respectively.

#### Perceived Stress Level

The Perceived Stress Scale (PSS) was used to measure perceived stress among study participants (47, 48). The PSS measures stress due to negative perception and lack of positive perception for a situation. Cronbach’s alphas were 0.818 and 0.827 for the first and second surveys, respectively.

#### Media Use

As reported previously, media exposure affects knowledge about COVID-19 and anxiety (23, 49). Therefore, the model for the moderating effects of knowledge about COVID-19 was analyzed with media use as a covariate. Previously described questionnaires (23, 49) that measure media and information use and exposure during COVID-19 were used. Cronbach’s alphas were 0.732 and 0.762 for the first and second surveys, respectively.

### Statistical Analysis

**FIGURE 1:**
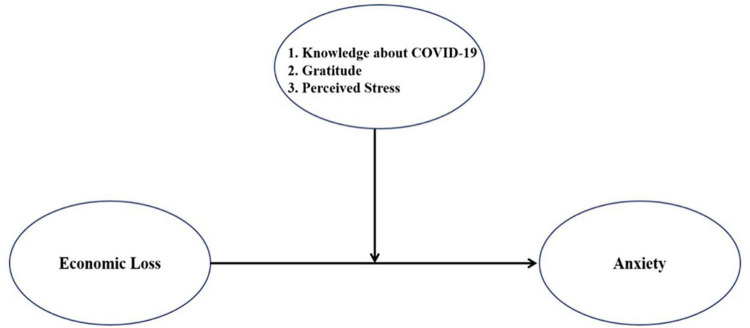
A model of the moderating effect.

SPSS Statistics 25.0 (IBM) and SPSS PROCESS Macro 3.5 (50) were used for analyses. PROCESS Macro is an analytical modeling tool proposed by Hayes in 2013 that enables the analysis of models, such as moderated, mediated, and adjusted-mediated models, using ordinary least squares and logistic regression (50). Descriptive statistics for individual characteristics and variables are presented as frequencies, percentages, means, and standard deviations. Pearson correlation analyses were used to assess correlations between primary and secondary variables. To examine the moderating effects of knowledge about COVID-19, gratitude, and perceived stress on the relationship between the independent variable (economic loss) and the dependent variable (anxiety), we used SPSS PROCESS Macro 3.5 (model 1: simple moderation model with one moderating variable) to verify the moderation model (50) (Figure 1). Johnson-Neyman analyses were conducted to probe trends in the interaction effects. Finally, data were analyzed to determine differences in moderating effects based on individual characteristics, such as sex, socioeconomic status, and religion.

## Results

### Sociodemographic Data

The 911 study participants included 466 males and 445 females. The average age of the respondents to the first survey was 41.5 years (*SD*: ± 11.7), 582 (63.9%) were regular workers/long-term contract workers, and 329 (36.1%) were short-term contract workers or those without any regular income (part-time workers, the unemployed, housewives, students, etc.). A total of 871 participants (95.6%) were covered by national health insurance, whereas 40 (4.4%) were recipients of medical aid or near-poverty individuals who earned 50% less than the standard median income. Frequencies, means, and standard deviations of key variables by sociodemographic group are presented in Table 2.

**TABLE 2:** Frequencies, means, and standard deviations of key variables by sociodemographic group (*N* = 911).

Variable		1st total (%)		M	SD	2nd total (%)		M	SD
Gender	Male	466 (51.1)	Economic loss (X1)	6.60	2.25		Economic loss (X2)	6.50	2.05
			Knowledge about COVID-19 (M11)	4.87	1.07		Knowledge about COVID-19 (M21)	4.65	1.15
			Media use (Covariate1)	13.25	2.87		Media use (Covariate2)	12.98	2.96
			Gratitude (M12)	29.42	5.95		Gratitude (M22)	28.64	5.66
			Perceived Stress (M13)	19.99	5.18		Perceived Stress (M23)	19.75	4.95
			Anxiety (Y1)	3.97	4.46		Anxiety (Y2)	3.89	4.20
	Female	445 (48.8)	X1	6.87	2.16		X2	6.53	2.12
			M11	5.05	1.02		M21	4.90	0.98
			Covariate1	14.22	2.66		Covariate2	13.94	2.65
			M12	30.41	5.75		M22	29.96	5.65
			M13	21.69	5.33		M23	21.26	5.32
			Y1	3.97	4.46		Y2	4.29	4.25
Age (±SD)		41.5 years (± 11.7)							
Religion	Presence	358 (39.3)	X1	6.92	2.18	349 (38.3)	X2	6.16	2.10
			M11	4.96	1.03		M21	4.72	1.10
			Covariate1	14.03	2.67		Covariate2	13.64	2.67
			M12	30.82	5.90		M22	29.43	5.61
			M13	20.69	4.93		M23	19.92	5.12
			Y1	3.83	4.34		Y2	3.77	4.06
	Absence	553 (60.7)	X1	6.61	1.07	562 (61.7)	X2	7.15	1.91
			M11	4.97	2.22		M21	4.80	1.06
			Covariate1	13.53	2.88		Covariate2	13.33	2.95
			M12	29.31	5.78		M22	29.02	5.82
			M13	20.90	5.56		M23	21.52	5.14
			Y1	4.31	4.41		Y2	4.56	4.46
Job type	Regular/	582 (63.9)	X1	6.39	2.20	586 (64.3)	X2	6.16	2.10
	Long-term		M11	4.96	1.05		M21	29.43	5.61
			Covariate1	13.47	2.70		Covariate2	13.35	2.86
			M12	29.90	5.77		M22	4.73	1.07
			M13	20.17	5.10		M23	19.92	5.12
			Y1	3.76	4.18		Y2	3.77	4.06
	Short-term	329 (36.1)	X1	7.35	2.10	325 (35.7)	X2	7.15	1.91
	contract/		M11	4.97	1.05		M21	4.84	1.08
	No income		Covariate1	14.17	2.94		Covariate2	13.62	2.83
			M12	29.91	6.06		M22	29.02	5.82
			M13	21.96	5.52		M23	21.52	5.14
			Y1	4.75	4.67		Y2	4.56	4.46
Socio	Medical	871 (95.6)	X1	6.70	2.22	868 (95.3)	X2	6.50	2.08
economic	insurance		M11	4.98	1.04		M21	4.80	1.06
status			Covariate1	13.72	2.81		Covariate2	13.48	2.86
			M12	30.03	5.81		M22	29.40	5.64
			M13	20.78	5.37		M23	20.47	5.17
			Y1	4.04	4.34		Y2	4.00	4.18
	Medical aid	40 (4.4)	X1	7.45	1.91	43 (4.7)	X2	6.77	2.20
			M11	4.48	1.22		M21	4.09	1.19
			Covariate1	13.93	2.83		Covariate2	12.91	2.54
			M12	27.25	6.54		M22	26.95	6.16
			M13	21.55	4.04		M23	20.86	5.57
			Y1	5.90	5.10		Y2	5.19	4.91

### Correlation Between Key Variables

First the correlation between the independent variable and the dependent variable was examined. Economic loss and anxiety were positively correlated in the first (*r* = 0.253, *p* < 0.01) and second (*r* = 0.259, *p* < 0.01) surveys. Next correlations between the dependent variable and the moderating variables were examined. Knowledge about COVID-19 (*r* = –0.251, *p* < 0.01) and gratitude (*r* = –0.289, *p* < 0.01) were negatively correlated with anxiety in the first survey, whereas perceived stress (*r* = 0.547, *p* < 0.01) was positively correlated. Similarly, knowledge about COVID-19 (*r* = –0.245, *p* < 0.01) and gratitude (*r* = –0.311, *p* < 0.01) were negatively correlated with anxiety in the second survey, whereas perceived stress (*r* = 0.520, *p* < 0.01) was positively correlated. Means, standard deviations, and correlation coefficients of key variables are presented in Table 3.

**TABLE 3:** The means, standard deviations, and correlation coefficients of the variables included in the moderation models.

	M	SD	1	2	3	4	5	6	7	8	9	10
1. Economic loss (1)	6.73	2.21	1									
2. Economic loss (2)	6.52	2.08	0.676**	1								
3. Knowledge about COVID-19 (1)	4.96	1.05	−0.091**	−0.114**	1							
4. Knowledge about COVID-19 (2)	4.77	1.08	−0.058	−0.088**	0.519**	1						
5. Gratitude (1)	29.90	5.87	−0.042	−0.073*	0.171**	0.178**	1					
6. Gratitude (2)	29.28	5.69	−0.079*	−0.093**	0.175**	0.163**	0.627**	1				
7. Perceived stress (1)	20.82	5.32	0.296**	0.247**	−0.100**	−0.076*	−0.298**	−0.228**	1			
8. Perceived stress (2)	20.49	5.18	0.249**	0.296**	−0.064	−0.059	−0.243**	−0.295**	0.596**	1		
9. Anxiety (1)	4.12	4.39	0.253**	0.231**	−0.251**	−0.246**	−0.289**	−0.272**	0.547**	0.445**	1	
10. Anxiety (2)	4.05	4.22	0.219**	0.259**	−0.191**	−0.245**	−0.222**	−0.311**	0.430**	0.520**	0.599**	1

**p < 0.01, *p < 0.05.

### The Relationship Between Economic Loss and Anxiety

We divided the repeatedly measured data into first and second surveys and examined the moderating effects of knowledge about COVID-19 (M11, M12), gratitude (M12, M22), and perceived stress (M13, M23) on the relationship between economic loss (X1, X2) and anxiety (Y1, Y2). In the moderation analyses, all variables were centered at their means.

### Moderating Effects of Knowledge About Coronavirus Disease 2019

Knowledge about COVID-19 did not moderate the relationship between economic loss and anxiety due to COVID-19 in the first (*B* = 0.0997, *p* = 0.123) or second (*B* = 0.0494, *p* = 0.416) survey (Table 4).

**TABLE 4:** The moderating effects of knowledge about COVID-19 on the relationship between economic loss and anxiety.

Variables	Anxiety (X1, X2)					
	*B*	SE	*t*	*p*	95% CI	
					LLCI	ULCI
Constant	0.2397	0.7012	0.3418	0.7326	−1.1365	1.6158
Economic loss (X1)	0.3508	0.0640	5.4846	0.0000	0.2253	0.4763
Knowledge about COVID-19 (M11)	−0.9443	0.1317	−7.1708	0.0000	−1.2028	−0.6859
X1 * M11	0.0997	0.0646	1.5435	0.1231	−0.0271	0.2265
Media use (covariate)	0.2841	0.0502	5.6601	0.0000	0.1856	0.3826
	*R^2^* = 0.1478, Adjusted *R*^2^ = 0.0022, *F*_(4, 906)_ = 39.2723, *p* < 0.000					

Constant	0.8404	0.6551	1.2828	0.1999	−0.4454	2.1261
Economic loss (X2)	0.3867	0.0657	5.8866	0.0000	0.2578	0.5156
Knowledge about COVID-19 (M21)	−0.8645	0.1222	−7.0764	0.0000	−1.1043	−0.6248
X2 * M21	0.0494	0.0607	0.8145	0.4156	−0.0697	0.1685
Media use (covariate)	0.2395	0.0477	5.0150	0.0000	0.1457	0.3332
	*R^2^* = 0.1411, Adjusted *R*^2^ = 0.0006, *F*_(4, 906)_ = 37.2155, *p* < 0.000					

*SE indicates standard error; LLCI and ULCI indicate confidence intervals; All variables were centered at their means; The models for each survey were tested independently.*

### Moderating Effects of Gratitude

**FIGURE 2:**
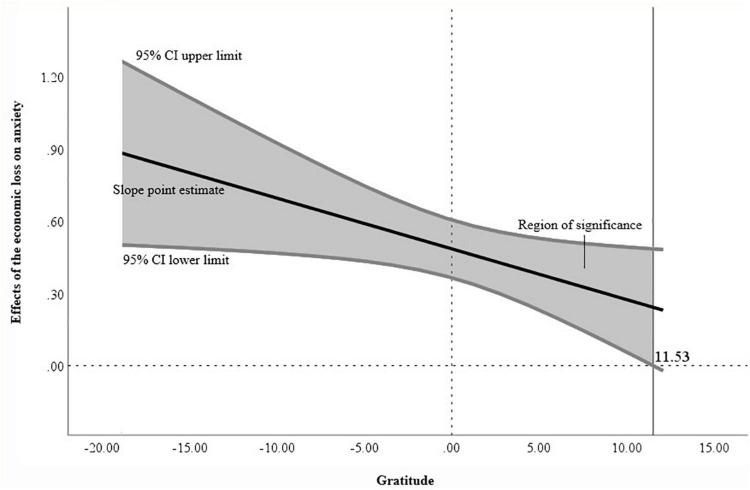
Johnson-Neyman analysis graph of the moderation effect of gratitude in first survey.

In the first survey, gratitude moderated the relationship between economic loss and anxiety due to COVID-19 (*B* = –0.0211, *p* < 0.05). The Johnson-Neyman method was used to probe the interaction and revealed that when the section of gratitude in the first survey was smaller than 11.5254, the coefficient between economic loss and anxiety was statistically significant (Figure 2). This indicates that lower levels of gratitude were associated with greater effects of economic loss on anxiety in the first survey. However, in the second survey, gratitude was not a significant moderating variable in the relationship between economic loss and anxiety due to COVID-19 (*B* = –0.0088, *p* = 0.3921). Statistics for the main analyses are presented in Table 5.

**TABLE 5:** The moderating effects of gratitude on the relationship between economic loss and anxiety.

Variables	Anxiety (X1, X2)					
	*B*	SE	*t*	*p*	95% CI	
					LLCI	ULCI
Constant	4.1060	0.1346	30.7016	0.0000	3.8419	4.3701
Economic loss (X1)	0.4851	0.0610	7.5713	0.0000	0.3653	0.6048
Gratitude (M12)	−0.2073	0.0229	−7.1696	0.0000	−0.2524	−0.1623
X1 * M12	−0.0211	0.0096	0.6894	0.0285	−0.0399	−0.0022
	*R^2^* = 0.1462, Adjusted *R^2^* = 0.0045, *F*_(3, 907)_ = 51.7785, *p* < 0.000					

Constant	4.0419	0.1297	31.1751	0.0000	3.7874	4.2963
Economic loss (X2)	0.4752	0.0625	7.6073	0.0000	0.3526	0.5978
Gratitude (M22)	−0.2140	0.0228	−9.3694	0.0000	−0.2588	−0.1692
X2 * M22	−0.0088	0.0103	−0.8562	0.3921	−0.0290	0.0114
	*R^2^* = 0.1509, Adjusted *R^2^* = 0.0007, *F*_(3, 907)_ = 53.7273, *p* < 0.000					

*SE indicates standard error; LLCI and ULCI indicate confidence intervals; All variables were centered at their means; The models for each survey were tested independently.*

### Moderating Effects of Perceived Stress

**FIGURE 3:**
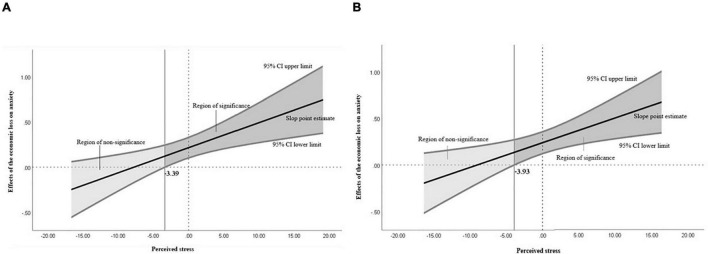
Graphs of the moderation effect of perceived stress. **(A)** Johnson-Neyman analysis graph in the first survey; **(B)** Johnson-Neyman analysis graph in the second survey.

In the first survey, perceived stress moderated the relationship between economic loss and anxiety due to COVID-19 (*B* = 0.0278, *p* < 0.01). Using the Johnson-Neyman method to probe the interaction, we found that when the section of perceived stress in the first survey was larger than –3.3860, the coefficient between economic loss and anxiety was statistically significant (Figure 3A). This indicates that the effect of economic loss on anxiety increased as perceived stress increased. Similarly, in the second survey, perceived stress significantly moderated the relationship between economic loss and anxiety (*B* = 0.0265, *p* < 0.01). When the section of perceived stress was greater than –3.9338 in the second survey, the coefficient between economic loss and anxiety was statistically significant (Figure 3B). This indicates that the effect of economic loss on anxiety increased as perceived stress increased. Statistics for the main analyses are presented in Table 6.

**TABLE 6:** The moderating effects of perceived stress on the relationship between economic loss and anxiety.

Variables	Anxiety (X1, X2)					
	*B*	SE	*t*	*p*	95% CI	
					LLCI	ULCI
Constant	4.0419	0.1297	31.1751	0.0000	3.7874	4.2963
Economic loss (X1)	0.2161	0.0574	3.7656	0.0002	0.1035	0.3288
Perceived Stress (M13)	0.4295	0.0237	18.0939	0.0000	0.3829	0.4761
X1 * M13	0.0278	0.0091	3.0519	0.0023	0.0099	0.0456
	*R^2^* = 0.3149, Adjusted *R^2^* = 0.0070, *F*_(3, 907)_ = 138.9434, *p* < 0.000					

Constant	3.9669	0.1220	32.5198	0.0000	3.7275	4.2063
Economic loss (X2)	0.2397	0.0595	4.0312	0.0001	0.1230	0.3563
Perceived Stress (M23)	0.4011	0.0240	16.7418	0.0000	0.3540	0.4481
X2 * M23	0.0265	0.0094	2.8059	0.0051	0.0080	0.0450
	*R^2^* = 0.2889, Adjusted *R^2^* = 0.0062, *F*_(3, 907)_ = 122.8199, *p* < 0.000					

*SE indicates standard error; LLCI and ULCI indicate confidence intervals; All variables were centered at their means; The models for each survey were tested independently.*

### Summary of Main Analyses

Moderating effects of knowledge about COVID-19, gratitude, and perceived stress on the relationship between economic loss and anxiety due to COVID-19 were verified. In the first survey, knowledge about COVID-19 had no moderating effect on the relationship between economic loss and anxiety due to COVID-19, but both gratitude and perceived stress had moderating effects. Conversely, in the second survey, only perceived stress had moderating effects.

### Differences in Moderating Effects by Individual Characteristics

Next we examined whether moderating effects varied by sex, religion, health insurance coverage, or job type. Among males, gratitude (*B* = –0.0304, *p* < 0.05) and perceived stress (*B* = 0.0462, *p* < 0.001) in the first survey and knowledge about COVID-19 (*B* = 0.1574, *p* < 0.05) in the second survey were significant moderating variables. This indicates that lower levels of gratitude and higher perceived stress increased the effect of economic loss on anxiety in the first survey. Meanwhile, greater knowledge about COVID-19 reduced the effect of economic loss on anxiety in the second survey. Among females, none of the three variables had moderating effects in the first survey, but the moderating effect of perceived stress (*B* = 0.0320, *p* < 0.05) was significant in the second survey. This indicates that greater perceived stress increased the effect of economic loss on anxiety in the second survey.

Among participants who reported the presence of religion, gratitude (*B* = –0.0316, *p* < 0.001) was the only variable with a significant moderating effect in the first survey. However, no moderating effect of gratitude was found in the second survey. Meanwhile, perceived stress (*B* = 0.0546, *p* < 0.001) was the only moderating variable in the second survey in this group. In the absence of religion group, the moderating effects of knowledge about COVID-19 (*B* = 0.1675, *p* < 0.05) and perceived stress (*B* = 0.0332, *p* < 0.01) were significant in the first survey, although there were no moderating variables in the second survey.

In terms of job type, the moderating effects of perceived stress in the first (*B* = 0.0251, *p* < 0.05) and second (*B* = 0.0421, *p* < 0.001) surveys were significant for participants who were regular or long-term contract workers. However, there were no significant moderating variables for non-regular workers or for those who had no regular income.

Among those who were covered by health insurance, the moderating effects of gratitude (*B* = –0.0209, *p* < 0.05) and perceived stress (*B* = 0.0278, *p* < 0.01) were significant in the first survey, although perceived stress (*B* = 0.0252, *p* < 0.01) was the only significant factor in the second survey. There were no significant moderating variables among recipients of medical aid.

## Discussion

This study examined the effects of knowledge about COVID-19, gratitude, and perceived stress on COVID-19-related anxiety in the public. We conducted two independent analyses using panel data obtained in April and May 2020 and in November 2020. Gratitude and perceived stress had moderating effects in the first survey (April and May 2020), but only perceived stress had a moderating effect in the second survey (November 2020). Moreover, analyses were conducted based on sex, religion, and socioeconomic status to determine whether different groups were affected differently by knowledge about COVID-19, gratitude, and perceived stress.

Gratitude and perceived stress showed moderating effects on the relationship between economic loss and anxiety in the first survey. In other words, in the early stages of COVID-19, lower levels of gratitude and higher perceived stress led to greater anxiety. These results are consistent with previous studies that have reported gratitude as a protective factor (29) and perceived stress as a risk factor (35). However, there were no moderating effects in groups with high levels of gratitude or those with low perceived stress. This is because anxiety is closely associated with the predictability and controllability of events. Economic loss can have significant direct and indirect effects on individuals’ survival. However, given the unpredictable nature of the COVID-19 pandemic, perceptions of predictability and controllability were reduced. Therefore, it is possible that gratitude decreased and anxiety increased with an increase in perceived stress.

Only perceived stress had a continued moderating effect on the relationship between economic loss and anxiety in the second survey. We observed an increase in anxiety as perceived stress increased. Given that this study was conducted repeatedly among the same participants, this indicates that gratitude, which was a protective factor in the first survey, had reduced moderating effects over time. This may be because people had different experiences at the time of the first survey, which was 3 months after the onset of COVID-19, corresponding to the honeymoon phase (3–6 months after a disaster) of the emotional phases of disasters, compared to the second survey, which was conducted when 6 months had passed. In the honeymoon phase of a disaster, national and local governments promise damage recovery and support and provide survivors with the hope of resources to rebuild their lives (51, 52). Therefore, the expected provision of resources by the government may have led the participants to be optimistic about economic loss and eventual recovery. However, the prolonged COVID-19 pandemic led to an increase in unemployment and absenteeism (3), thereby gradually increasing the economic deficit experienced by individuals (2, 3). Because of the longevity of the pandemic, individuals continued to experience physical and emotional fatigue. By the end of the honeymoon phase (8 months to 2 years after a disaster), individuals become less hopeful for the restoration and recovery of life and become increasingly distrustful of government support (52). During this phase, positive emotions (such as hope and relief), the predictability of events, and the sense of control felt at the beginning of the pandemic may have decreased (52). However, anxiety did not decrease as the COVID-19 pandemic continued. It is likely that at the time of the second survey, when the pandemic had become prolonged, gratitude, as a spiritual coping method or personality trait, could not act as a protective factor against economic loss or stress. Previous studies have reported a temporal decline in mental health during epidemics (53, 54) that tends to persist even after the epidemic has ended (55, 56). However, there are limitations to what individuals can do to improve their coping skills. Psychological support should go hand in hand with effective systems for taxation, debt, management support, job creation, and so forth. Moreover, it is important to promote mental health at the community level using psychosocial interventions. Therefore, the government and local communities should take measures to provide sustainable help.

It is necessary to look at the economic losses brought about by the spread of COVID-19 in socioeconomic and political contexts. Since the 1997 Asian foreign exchange crisis, concerns about polarization as a side effect of rapid economic growth have been constantly raised in South Korea (57–59). The prolonged spread of COVID-19 has made the low-income class more economically vulnerable, further exacerbating economic inequalities and polarization (60). In the midst of an economic crisis that is hard even for individuals considered persistent to cope with, the unpredictable economic loss and the lack of trust in protective measures by the government can have significant adverse effects on individuals’ mental health (61). In a survey conducted by the Ministry of Health and Welfare in South Korea (62), the majority of people agreed with the effectiveness and need for social distancing but were doubtful about the fairness of its implementation (63) or the sufficiency of the state’s financial support. It seems appropriate for future studies to consider the effects of confidence or trust among individuals who have faced economic loss in the pandemic and the mental health support that can be provided by the environment.

It is also interesting that there were no significant moderating effects of knowledge about COVID-19 in our main study, although the group-specific analyses found moderating effects among males and those with an absence of religion. This contrasts with earlier studies (24) that have reported that knowledge about infectious diseases reduces anxiety during pandemics. These findings can be explained by the ambiguity of the variable “acquisition of information.” Although appropriate and accurate knowledge reduces anxiety and confusion during a pandemic (23, 24), acquiring knowledge through stimulating media reports increases distorted perceptions along with uncertainty about the pandemic and may further amplify anxiety and fear among individuals (64). Therefore, governments and communities should ensure the prompt availability of accurate information.

In addition, the COVID-19 pandemic is different from previous SARS coronavirus and Middle East Respiratory Syndrome (MERS) epidemics. As new variants continue to emerge, disparities in vaccine rollout procedures across countries and uncertainties about side effects of vaccines can lead to disability and death. The unexpectedness and unpredictability of the current pandemic separates it from previous infectious diseases. It is possible that the protective effects of knowledge about the pandemic and gratitude reported in previous studies were absent in COVID-19. In fact, when stress levels due to COVID-19 among South Koreans were compared to those due to other disasters in Korea, they were 1.5 times those of the MERS outbreak and 1.4 times those of the Gyeongju and Pohang earthquakes (53). Therefore, the emotional distress caused by the COVID-19 pandemic is more severe than that caused by other disasters.

Perceived stress had a moderating effect among males in the first survey; among females in the second survey; among those with full health insurance coverage, regular jobs, and religion in the second survey; and among those with no religion in the first survey. This suggests that, in addition to the strong stressor effects of COVID-19, individual perceptions of the economic situation during COVID-19 are also risk factors that can affect anxiety over time. Among males, moderating effects appeared at the beginning of the pandemic, and greater perceived stress caused higher levels of anxiety. However, the moderating effects disappeared 6 months later when the pandemic had become prolonged. These results may indicate that perceived stress levels may act as a risk factor in the early stage of a disaster among males, but they may be more adaptive to prolonged stress. Conversely, among females, there were no protective factors in the early stages of the pandemic, but perceived stress acted as a risk factor in later stages of the prolonged pandemic (i.e., females may be more vulnerable to long-term disasters). In addition, given that perceived stress continued to act as a risk factor in groups that were less likely to be affected by catastrophic expenditures for health care services (regular workers and those with health insurance coverage), and in both the religion and non-religion groups, perceived stress appears to be a major health risk for all individuals at all times. Therefore, psychological interventions to reduce perceived stress seem necessary.

The study has some limitations. As this study involved the use of cross-sectional surveys at two different time points, it is difficult to make longitudinal interpretations of patterns of change in variables over time, causal relationships, and interactions between variables. In the future, longitudinal studies are required to use multivariate latent growth modeling to determine the change in variables over time, to verify relationships between these changes, and to analyze individual differences in these changes. Moreover, it may be necessary to obtain data at fixed intervals while considering the duration and severity of the pandemic, government policies, and major events to determine patterns of change and causal relationships. Furthermore, this study focused on generalized anxiety only. Instruments and questionnaires for anxiety other than the GAD-7 may be used to explore and compare specific types of anxiety (social anxiety, health anxiety, agoraphobia, etc.). Although perceived stress was the only consistent moderator at the beginning of the outbreak and afterward, it would be worth exploring the effects of ecological variables, such as government financial support and social distancing policies, as economic problems can be intertwined at the individual and structural levels. We used medical insurance to measure individual socioeconomic status in this study, but there were large differences in sample sizes by insurance group because of the nature of national insurance system in South Korea. According to the National Statistical Portal of South Korea (65), in 2020, a total of 51,344,938 individuals received medical benefits, whereas the number of individuals in low-income and near-poverty groups who received medical aid was 1,526,030, accounting for only 3% of the population. The classification of 4.4% of the population into the low-income group in this study accurately reflected the population in its own way. However, the sample size in this group was relatively small, and the definition of low income could vary in different countries. Therefore, the findings for the low-income group should be interpreted cautiously.

## Data Availability Statement

The raw data supporting the conclusions of this article will be made available by the authors, without undue reservation.

## Ethics Statement

The studies involving human participants were reviewed and approved by the Chonnam National University Hospital Institutional Review Committee (CNUH-2020-092). The patients/participants provided their written informed consent to participate in this study.

## Author Contributions

HJ, Y-SK, S-WK, A-LP, Y-RL, SR, J-YL, and J-MK analyzed and interpreted the data. S-WK designed and collected the data. HJ and Y-SK wrote the manuscript. All authors approved the final version of the article.

## Conflict of Interest

The authors declare that the research was conducted in the absence of any commercial or financial relationships that could be construed as a potential conflict of interest.

## Publisher’s Note

All claims expressed in this article are solely those of the authors and do not necessarily represent those of their affiliated organizations, or those of the publisher, the editors and the reviewers. Any product that may be evaluated in this article, or claim that may be made by its manufacturer, is not guaranteed or endorsed by the publisher.
